# Defining Natural Antibodies

**DOI:** 10.3389/fimmu.2017.00872

**Published:** 2017-07-26

**Authors:** Nichol E. Holodick, Nely Rodríguez-Zhurbenko, Ana María Hernández

**Affiliations:** ^1^Department of Biomedical Sciences, Center for Immunobiology, Western Michigan University Homer Stryker M.D. School of Medicine, Kalamazoo, MI, United States; ^2^Natural Antibodies Group, Tumor Immunology Division, Center of Molecular Immunology, Havana, Cuba

**Keywords:** natural antibody, antibodies, natural antibody repertoire, B-1 cells, B cell subsets, B cells

## Abstract

The traditional definition of natural antibodies (NAbs) states that these antibodies are present prior to the body encountering cognate antigen, providing a first line of defense against infection thereby, allowing time for a specific antibody response to be mounted. The literature has a seemingly common definition of NAbs; however, as our knowledge of antibodies and B cells is refined, re-evaluation of the common definition of Nabs may be required. Defining Nabs becomes important as the function of NAb production is used to define B cell subsets (1) and as these important molecules are shown to play numerous roles in the immune system (Figure 1). Herein, we aim to briefly summarize our current knowledge of NAbs in the context of initiating a discussion within the field of how such an important and multifaceted group of molecules should be defined.

## Natural Antibody (NAb) Producing Cells

Both murine and human NAbs have been discussed in detail since the late 1960s (2, 3); however, cells producing NAbs were not identified until 1983 in the murine system (4, 5). These cells, named B-1 cells, were originally identified by their expression of CD5 and were further characterized by surface expression of IgM^high^, IgD^low^, CD19^high^, B220^low^, CD23^−^, and CD43^+^ (6), which contrasts with the surface phenotype of follicular B-2 cells: CD5^−^, IgM^low^, IgD^high^, CD19^+^, B220^+^, CD23^+^, and CD43^−^. Later, an additional population of B-1 cells was identified, which shared the characteristics of CD5^+^ B-1 but lacked CD5 expression (7). These two populations of B-1 cells are termed B-1a (CD5^+^) and B-1b (CD5^−^) cells. B-1 cells also express CD11b; however, this expression is limited to B-1 cells residing in the body cavities and is lost upon migration to the spleen (8, 9). Furthermore, the B-1 cell population can be divided not only phenotypically but also functionally into natural or antigen-induced antibody secreting cells (10).

**Figure 1:**
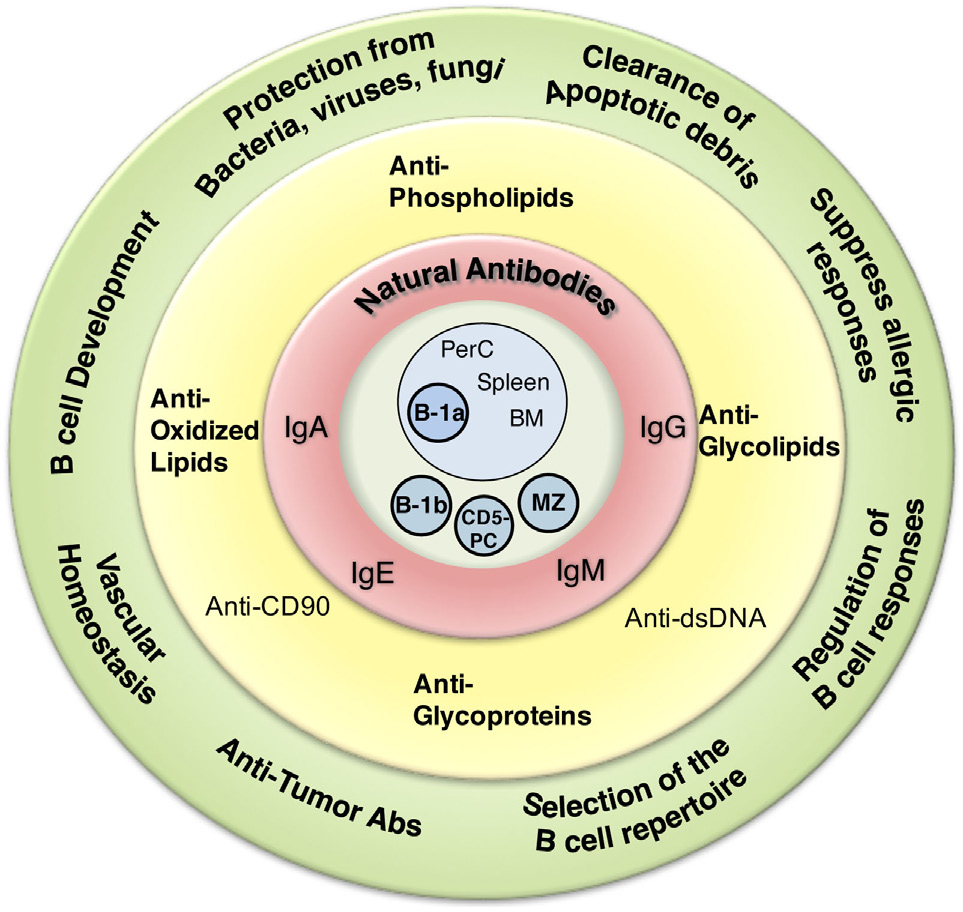
Overview of natural antibodies’ (NAbs) attributes. Graphical representation of the various NAb functions (outside green circle), epitope recognition (inside yellow circle), isotype (inside red circle), and cells shown to produce NAbs (inside blue circle).

B-1 cells are found in various tissues of adult mice, which include the peritoneal cavity, pleural cavity, spleen, bone marrow, lymph nodes, and blood [reviewed in Ref. (11)]. The tissue location may influence the functional role of B-1 cells. The peritoneal and pleural cavities have been shown to be an important reservoir for B-1 cells that respond to various stimuli (12–16) and subsequently migrate to the spleen/mesenteric or mediastinal lymph nodes, respectively, where they begin to secrete antibody (17). In mice depleted of B cells, peritoneal B-1 cells have the ability to fully reconstitute natural serum immunoglobulin (Ig) M as well as B-1 cells in all tissue locations (18); yet, in normal healthy mice, peritoneal B-1a cells do not directly contribute to natural serum IgM (19). Instead, the direct sources of natural serum IgM are B-1a cells located in the spleen and bone marrow (19). It has been shown that peritoneal B-1a cells recirculate from the peritoneum to the blood in a CXCL13-dependent manner (20). Interestingly, in the absence of CXCL13, mice are devoid of peritoneal B-1 cells but still have splenic B-1 cells; yet, despite having normal levels of serum IgM these mice have significantly less natural IgM specific for phosphorylcholine (20). This study suggests that it is possible for peritoneal B-1 cells to contribute to the splenic B-1 cell population and this recirculation might be particularly important for certain Nab reactivites. To date, the exact developmental relationship between the Nab secreting splenic/bone marrow B-1a cells and peritoneal B-1a cells is still unknown.

Beyond heterogeneity at different tissue sites, various subpopulations of B-1a cells have been defined based on surface marker expression. In the peritoneal cavity, B-1a subpopulations include PD-L2 (PD-L2^+/−^) (21, 22), CD25 (CD25^+/−^) (23), CD73 (CD73^hi/lo^) (24), and PC-1 (PC-1^hi/lo^). The PD-L2, CD25, and CD73 subsets showed no difference in the amount of natural IgM secretion between positive and negative subsets (21–24). Conversely, PC-1 B-1a cell subsets differed in the level of natural IgM secretion. PC-1^lo^ B-1a cells were shown to produce the large majority of natural IgM (25). PC-1^hi^ B-1a cells produced a significantly lower level of natural IgM and contained B-1a cells producing the antiphosphatidylcholine (anti-PtC) specificity (25, 26). B-1a cells have also been shown to produce IL-10 in the absence of stimulation (27); however, the relationship between regulatory (B10) cells (28) and B-1a cells is still unknown. In the bone marrow, a fetal-derived B cell subset was recently identified, which phenotypes as a plasmablast/plasma cell (CD5^−^IgM^+^IgD^−^CD138^+^B220^lo/−^FSC^hi^CD43^+^) (29); it is unknown whether this population is a terminally differentiated B-1, B-2, or novel population of cells (19). In the spleen, a population of CD138 + B-1a cells is present in unimmunized mice, which rapidly respond to stimulation prior to immigration of peritoneal B-1 cells to the spleen (30). The spleen is also home to marginal zone B cells, which also produce NAbs (31); however, it has been demonstrated that greater than 90% of Nab is produced by B-1 cells (18).

Given the evidence described previously, it is clear in mice, more than one B cell population is responsible for NAb production and not all subsets of B-1 cells spontaneously secrete NAbs that accumulate in serum. Thus, the generalization that all B-1 cells secrete NAbs should be avoided. This point has important implications when comparing the molecular repertoire of a certain B-1 cell subset as it relates to the total natural serum IgM repertoire, which would include the molecular repertoire of numerous B-1 cell subsets from various locations (10).

Natural antibody secreting cells in humans were first identified as CD5^+^ peripheral B cells (32–35). Later, it was demonstrated that CD5^−^CD45RA^lo^ peripheral B cells could also produce natural IgM (36). Much of the early work in humans focused on characterization and comparison of polyreactive antibodies, which were shown to utilize VH4 more frequently than monoreactive antibodies (37). More recently, strides have been made to refine the phenotypic characterization of Nab producing cells in the human system by starting with functional characteristics such as natural/spontaneous antibody secretion. This approach yielded a new phenotypic definition, CD20^+^CD27^+^CD43^+^CD70^−^CD38^mod^, of Nab secreting cells, the majority of which express CD5 (1, 38). Nevertheless, the phenotype of antibody secreting cells in the peripheral blood of humans is still evolving. Further investigation of Nab secreting cells in the human system is needed to elucidate the specific types of cells that are capable of producing NAbs, as well as the location of these cells beyond peripheral blood.

## NAb Reactivity

Although NAbs are known for their broad reactivity against self-antigens, some have the ability to recognize evolutionarily fixed epitopes present in foreign antigens. Whether or not NAb recognition of foreign structures is always the result of cross-reactivity against self-antigens is still a matter of debate. Generally, the most well-characterized epitopes to date include phospholipids, oxidized lipids, glycolipids, and glycoproteins. The best characterized B-1 cell-derived NAb binds the phospholipid phosphorylcholine and utilizes VHS107.1 (39). Phosphorylcholine is found within the bacterial cell wall of *Streptococcus pneumoniae* (40) and is also exposed on apoptotic cells and oxidized lipids (41–45). In normal healthy cells, phosphorylcholine is hidden within the head group of another well-characterized NAb epitope, PtC. PtC is a normal constituent of cell membranes, which is exposed upon treatment with the protease, bromelain (46–49). Early studies revealed NAb binding to red blood cells treated with bromelain were B-1 cell derived and utilized VH11 (50, 51), VH12 (52), and Q52 (53).

Antibodies that recognize glycan epitopes are also highly abundant in both mice and humans (54, 55). Glycan epitopes are observed on both glycoproteins and glycolipids and can be present in autologous or pathogen-associated exogenous structures. In mice, the specificities of such antibodies are thoroughly reviewed by New et al., which include alpha-1,3-glucan, *N*-acetyl-d-glucosamine, and alpha-1,3-galactose epitopes (56). In humans, the best known antiglycan antibodies react with blood group antigens A and B (57), the xenoantigen Gal-alpha-1,3Gal-beta-1,4GlcNAc (58, 59), Forssman glycolipid antigen, and gangliosides such as the tumor-associated antigen Neu5GcGM3 (60).

## NAb Functions

Natural antibodies provide various essential functions within the immune system. The most prevalently studied function is the ability to provide protection against bacterial, viral, and fungal infections. Such protection is afforded by Nabs’ epitope recognition. In particular, NAbs have been shown to provide protection against *S. pneumoniae* (61–63), sepsis (64), *Borrelia hermsii* (65), influenza virus (66), *Listeria monocytogenes* (67), vesicular stomatitis virus (67), lymphocytic choriomeningitis virus (67), *Cryptococcus neoformans* (68), and *Pneumocystis murina* (69). In addition to Nabs to the aforementioned organisms, B-1 cells produce “induced” antibody responses against *S. pneumoniae* (61), *B. hermsii* (65, 70, 71), influenza virus (12, 66, 72), and *Francisella tularensis* (13, 73).

Beyond protection against various infections, NAbs serve a number of other essential functions in the immune system. These functions have been reviewed extensively elsewhere (56) and include regulation of B cell development (10, 74, 75), selection of the B cell repertoire (74, 76), regulation of B cell responses (77), clearance of apoptotic debris (45), vascular homeostasis/protection against atherosclerosis (78–81), allergic suppression (82, 83), and protection from cancer (84, 85) (Figure 1). Despite this broad range of identified NAb functions, the role of NAbs in the immune system continues to expand.

## NAb Characteristics

In mice, typical characteristics of NAbs include germline-like nucleotide structure, repertoire skewing, IgM, IgA, or IgE (86) isotype, and T cell independence. Classically, NAbs are defined as being germline like as evidenced by these antibodies lacking non-templated nucleotides (N-additions) and having little to no somatic hypermutation (39, 87, 88). Antigen receptor diversity is increased during VDJ recombination when the enzyme TdT is present, which adds N-additions to the V-D and D-J junctions (89). Such germline characteristics have been shown to be essential in NAbs’ ability to protect against infection. The prototypical B-1a anti-phosphorylcholine antibody, T15, has no N-addition (90, 91). In mice with forced expression of TdT, all anti-PC antibodies generated after vaccination with heat killed *S. pneumoniae* contain N-additions; however, these anti-phosphorylcholine antibodies containing N-additions were shown to provide no protection against *S. pneumoniae* infection (92). This study highlights the importance of germline structure in the protection provided by evolutionarily conserved Nab. In addition, NAbs derived from murine B-1a cells have a restricted repertoire. On average 5–15% of peritoneal B-1a cells recognize PtC and utilize VH11 and VH12 (93).

Other studies have shown that these “classical” characteristics of NAbs do not always apply. For instance, B-1a cells from 6- to 24-month-old mice produce Igs with significantly more N-additions (94, 95). Furthermore, it was demonstrated that B-1a cells accumulate somatic hypermutations with increasing age, which is AID dependent (96). In this same study, isotype switching was also increased in B-1a cells with age (96). Nonetheless, throughout the decades of Nab investigation, IgG and IgA have been shown to be present within the NAb pool (97–99); however, natural IgG and IgA levels decrease significantly in germ-free mice, whereas IgM levels remain unaffected (100). This suggests the amount of natural serum IgG and IgA are dependent upon exogenous antigen stimulation, whereas the level of natural serum IgM is not.

In humans, studying NAbs in the absence of antigen exposure is a challenge; however, studies performed during early human life provide a period of limited exogenous antigen exposure in the presence of undistributed, strictly controlled intrauterine antigen milieu (101). It was demonstrated that inside the fetal B cell population at 12–14 weeks of human gestation, only IgM and IgD transcripts were detected (101). Yet, after 26 weeks of gestation, B cell clones encoding IgG start to appear in a frequency similar to a frequency observed in healthy infants, which suggests IgM is not the only isotype present in the prenatal repertoire of human B cells. Furthermore, somatic hypermutations occur during human fetal B cell development even in a T cell-independent fashion (101). As described in mice, early human NAbs are also diverse in isotype and structure.

Non-templated nucleotides (junctional diversity) are also an important mechanism of generating Ig structural diversity, which along with combinatorial diversity and somatic mutation results in numerous Ig specificities (102–104). In mice, natural B-1a cell-derived IgM is characterized by a low number of N-additions (105). Interestingly, TdT expression is restricted to adult life in mice (89), which is after the majority of fetal derived B-1a cell development has occurred (105, 106). Therefore, in mice, fetal-derived B-1a cells lack N-additions (106), whereas adult bone marrow-derived B-1a cells display a high level of N-additions (95, 107–109). In contrast, TdT is expressed during both fetal and adult life in humans, and as a result, both fetal and adult derived human B cells express Ig with numerous N-additions (110). Yet, it has been shown human and mouse fetal sequences share both similarities and differences in their repertories (111). For example, even though TdT is present throughout early human life, it has been demonstrated that the number of N-additions/CDR-H3 length in B cells from preterm and term infants are shorter than that of adults (112).

## Defining NAbs

As one reads through the body of NAb literature from the early 1960s to the present day, it becomes increasingly difficult to find a common concrete definition. The most frequently used definition describes NAbs as preimmune antibodies generated in the absence of exogenous antigenic stimulation, which are non-specific, broadly cross-reactive, low affinity, germline-like antibodies. As summarized in Figure 1, NAbs have many attributes, although NAbs cannot be defined by several of these characteristics. Furthermore, NAbs cannot be defined based on a single B cell subset or location. Different subsets of B cells in different locations are capable of secreting NAbs. Neither a specific isotype nor a specific function can define NAbs. Therefore, the characteristics left to define NAbs include how they are generated (presence or absence of endogenous and/or exogenous antigen) and their structural composition (germline-like or diverse).

In terms of specific reactivity to exogenous antigens, studies have indicated that B-1a cells in the peritoneal cavity serve as a long-term reservoir of “natural” antibody-producing cells after first exposure to the antigen (17). However, if these B-1 cells have previously seen their cognate antigen it might be more appropriate to term these as memory B-1 cells. In fact, some subsets of peritoneal B-1a cells share similarities to memory B cells such as PD-L2 and CD73 expression (21, 24). Thus, it has been suggested that within the B-1 cell population, those residing in the bone marrow and the spleen are the true Nab-secreting cells (17), whereas body cavity B-1 cells constitute a population of responder (memory type) lymphocytes, which after stimulation migrate and differentiate to IgM-secreting cells. As such, it is possible body cavity B-1 cells should not be considered NAb secretors since intentional stimulation is required to upregulate the secreting process.

Other studies indicate exogenous antigens are required for selection of the overall B cell repertoire (76). In addition, altering antigenic exposure during neonatal life has been shown to significantly change the repertoire of adult B cells (82). B-1a cells are generated mainly during the fetal/neonatal period; therefore, any antigen exposure during neonatal life would be expected to significantly influence the development of B-1a cell-derived NAbs. Interestingly, it has been suggested that the neonatal period is subject to increased intestinal permeability and this access point for antigen exposure could direct the development of NAbs (56). Nonetheless, no significant difference was observed between the B-1a cell derived IgM repertoire in germ-free mice when compared with specific pathogen-free mice (96, 98, 113, 114). In adult humans, the issue of antigen exposure is more of a problem as the antigenic exposure of humans cannot be controlled; therefore, studying a pre-immune repertoire is nearly impossible.

Schroeder and colleagues demonstrated the importance of both endogenous self-antigens and germline structure of antibodies in shaping the NAb repertoire. They showed the ability of the T15 NAb to clear endogenous antigen (oxidized low-density lipoprotein) is only dependent upon selection driven by self-antigens regardless of germline antibody structure, whereas the effectiveness of T15 to protect against exogenous antigen (phosphorylcholine/*S. pneumoniae*) is dependent upon both germline conservation and selection by self-antigen (115, 116). This is in line with studies by Kearney et al. demonstrating the influence of exogenous antigen upon the effectiveness of anti-phosphorylcholine antibody against *S. pneumonia* versus allergy (82). Together, these studies demonstrate how endogenous antigen, exogenous antigen, and germline composition create and alter the NAb repertoire.

Overall, these NAb studies call into question how Nabs can/should be accurately defined. Recently, this point has been plainly discussed in two separate reviews. The first by Baumgarth et al. (17), suggests an explicit definition: “we suggest the term *natural IgM production* be restricted to the truly antigen-independent elaboration of IgM in the spleen and bone marrow and not be extended to antigen-induced responses by B-1 cells.” The second by New et al. (56) states: “Thus, the generalization often made that the NAb repertoire develops independently of exogenous is not universal for all NAb specificities, and further research focusing on the factors contributing to the development and the composition of the NAb repertoire is warranted.” Herein, we suggest that these seemingly separate points of view can find common ground with further investigation.

**Figure 2:**
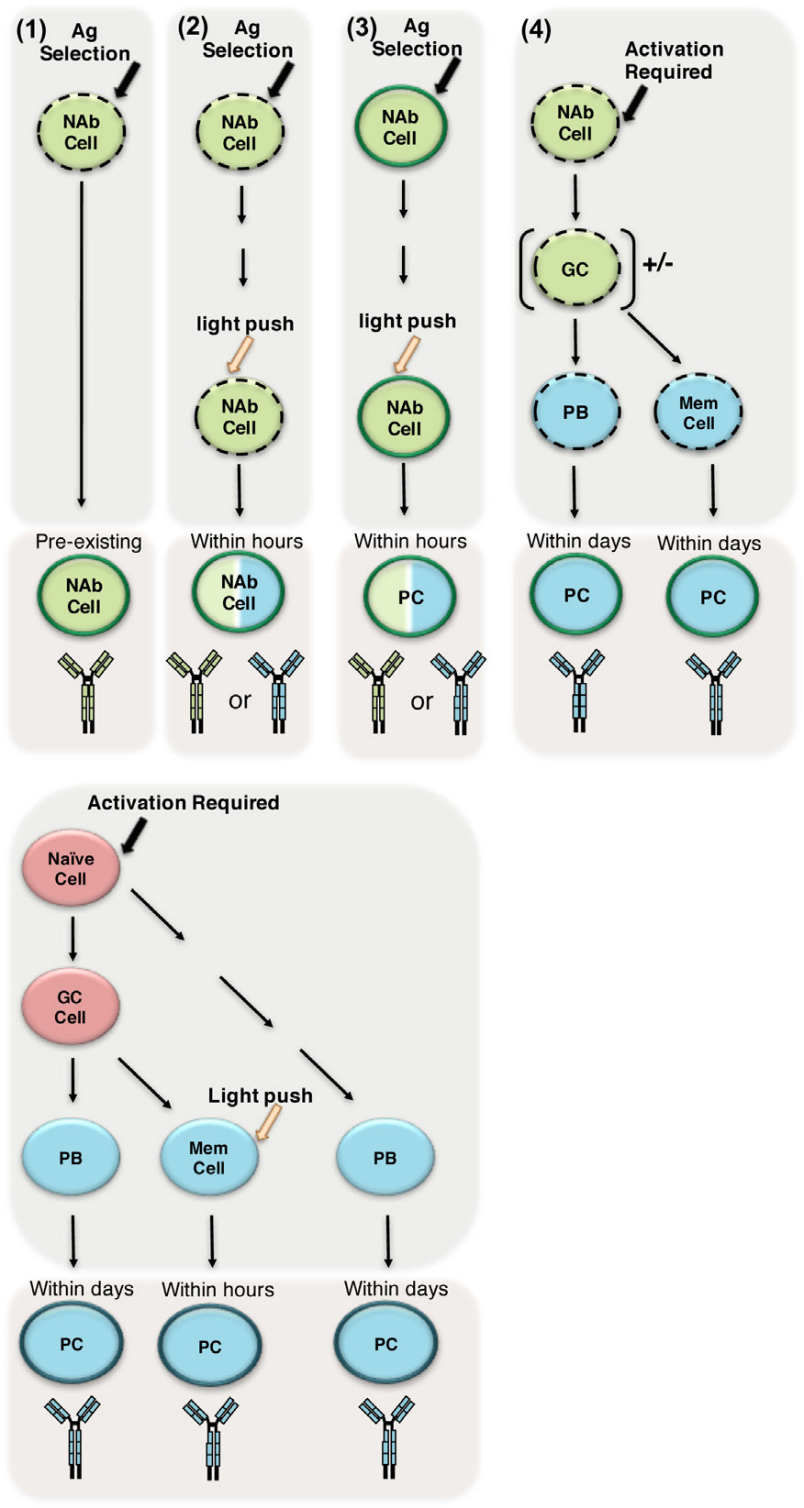
Determining how to define natural antibodies (NAbs). (1) Most frequently used definition of NAbs: preimmune antibodies generated in the absence of exogenous antigenic stimulation, which are broadly reactive, low affinity, germline-like antibodies selected in the presence of endogenous antigen (depicted in green). These antibodies are pre-existing/always present in the serum. The dotted outline represents the intrinsic properties the cell might have (e.g., increased levels of IgM and/or CD86/CD80, lower activation threshold). The dark outline indicates the cell is secreting Ig. (2–4) In panels 2–4, we suggest possible antigenic experiences of NAbs; however, further investigation is required to determine whether antibodies produced after such antigenic experiences are the same as preimmune, pre-existing NAbs in terms of germline structure and/or repertoire. (2) Here, we suggest a NAb producing cell may require an extra antigen experience (light push^(1)^) to start immediately (within hours) secreting. This antigen experience contrasts the strong activation required for naive B-2 cells (depicted in red) to differentiate into plasma cells (PC), which results in highly specific non-germline antibody (depicted in blue). However, it is unknown what affect this light push might have upon the antibody produced by the NAb producing cell (this is depicted by giving the cell both blue and green antibody colors). (3) We suggest a NAb producing cell that is already secreting NAbs experiences antigen, which induces differentiation into a plasma cell (PC). Again, it is unknown what affect this differentiation might have upon the antibody produced (this is depicted by giving the cell both blue and green antibody colors). (4) Finally, we depict NAb producing cells experiencing strong/specific activation and subsequently following the traditional pathway leading to memory and plasma cell differentiation.

It is clear a NAb repertoire can be created in the absence of exogenous antigens and/or germinal center maturation, and perhaps this is the definition of NAbs in its purest form. Yet, it has also been demonstrated that NAbs are affected by the presence of exogenous antigen, which is encountered in normal functioning systems. As an attempt to incorporate the role of antigen in the NAb repertoire, we propose the following starting point for investigation. To be a NAb, two requirements are necessary: (1) the ability to exert a protective, regulatory, or other biological function and (2) pre-existing/immediately responsive antibody. In the first requirement, the biological function might be protective, regulatory, or provide a function yet to be elucidated. In the second requirement, the antibody must already be present and secreted, or the NAb encoding cell would need only a light push^1^ for the NAb to be secreted.^2^ The role of antigen comes into play when considering the light push that some NAb secreting cells might need to immediately produce antibody. Furthermore, the ability of the NAb encoding cell to respond to the light push would be dependent upon its intrinsic properties such as status of surface phenotype or activation threshold. Further experimentation is required to determine whether the NAb produced by NAb secreting cells needing antigen exposure to immediately produce antibody differs from antibodies produced by other cells capable of immediate production of antibody (i.e., memory cells) (Figure 2). It is these authors perspective, as a field studying this clearly essential part of the immune system, we need to further investigate all contexts in which NAbs are produced and regulated (Figure 2).

## Author Contributions

All authors contributed to the writing and development of the perspective put forth. All authors worked together to edit and revise the manuscript.

## Conflict of Interest Statement

The authors declare that the research was conducted in the absence of any commercial or financial relationships that could be construed as a potential conflict of interest.

## Footnotes

^1^We are suggesting a light push to mean exposure to antigen or other stimuli leading to a differentiation pathway operating outside of the strong stimuli provided by the germinal center environment and thus, different from the naive B-2 cell requirement for stimulation.

^2^The subset of Nab-producing cells needing a light push might be poised to secrete but are not detected as such by the methods currently available.
